# Effects of Body Condition and Concentrate Proportion of the Ration on Mobilization of Fat Depots and Energetic Condition in Dairy Cows during Early Lactation Based on Ultrasonic Measurements

**DOI:** 10.3390/ani9040131

**Published:** 2019-03-29

**Authors:** Katharina Bünemann, Dirk von Soosten, Jana Frahm, Susanne Kersten, Ulrich Meyer, Jürgen Hummel, Annette Zeyner, Sven Dänicke

**Affiliations:** 1 Institute of Animal Nutrition, Friedrich-Loeffler-Insitut (FLI), Federal Research Institute for Animal Health, 38116 Braunschweig, Germany; Katharina.Buenemann@fli.de (K.B.); Jana.Frahm@fli.de (J.F.); Susanne.Kersten@fli.de (S.K.); Ulrich.Meyer@fli.de (U.M.); Sven.Daenicke@fli.de (S.D.); 2 Department of Animal Sciences, University of Goettingen, 37077 Goettingen, Germany; jhummel@gwdg.de; 3 Institute of Animal Nutrition, Martin Luther University Halle-Wittenberg, 06120 Halle (Saale), Germany; annette.zeyner@landw.uni-halle.de

**Keywords:** dairy cows, animal health, body condition score, concentrate feed proportion, energy metabolism, lipid mobilization, postpartal period

## Abstract

**Simple Summary:**

During early lactation, cows face metabolic challenges. They experience a negative energy balance as energy intake increases more slowly than energy output with milk rises. To compensate for that energy deficit, higher amounts of concentrate are offered. Additionally, cows are able to extract energy from body fat by lipid mobilization. Excessive body fat mobilization, however, leads to metabolic disorders. Therefore, high-conditioned cows are suggested to have a more pronounced lipid mobilization. The intention of the present study was to examine the change of various fat depots during the transition period depending on body condition and energy supply with ultrasonic measurements. Body condition loss after calving usually interpreted as mobilization of subcutaneous adipose tissue was not different between cows with a higher or lower body condition score. However, ultrasonic measurements detected a more pronounced mobilization of subcutaneous adipose tissue in higher conditioned animals. In contrast, inner fat depots were mobilized similarly between cows. Higher concentrate feed proportions led to a less pronounced negative energy balance. A less pronounced negative energy balance would have been expected to decrease lipid mobilization. However, this relation could not be verified in the present study. This demonstrates that sonography-based methods provide a clearer picture of metabolic conditions.

**Abstract:**

The aim of this study was to evaluate energy metabolism and lipid mobilization via ultrasonic measurements (USM), considering inner fat depots, in lactating dairy cows differing in body condition score (BCS) and fed rations with low (35% at dry matter basis; C_35_) or high (60% at dry matter basis; C_60_) concentrate feed proportions postpartum. Sixty pluriparous German Holstein cows were arranged in a 2 × 2 factorial design from d 42 antepartum (relative to calculated calving) until d 120 postpartum. Animals were divided into a group with a lower (initial BCS = 3.1 ± 0.38 SD; BCS_L_) and a group with a higher (initial BCS = 3.83 ± 0.41 SD; BCS_H_) BCS. Due to higher dry matter intake C_60_ groups reached the positive energy balance earlier, whereas C_35_ groups had a more pronounced negative energy balance. Although this would suggest a more pronounced mobilization of C_35_ groups the USM revealed no differences between feeding groups. Differences in BCS between both BCS groups remained almost the same over the trial. This was not reflected in ultrasonic data, as lipid mobilization was higher in higher conditioned cows. These findings demonstrate the extended possibilities of USM to depict metabolic processes.

## 1. Introduction

Cows are exposed to metabolic challenges during the transition period. The imbalance between an insufficient increase of energy intake on the one hand and the high energy requirements, on the other hand, induce a negative energy balance [1,2]. The organism attempts to compensate the energy deficit by mobilizing energy from adipose tissue depots [3]. This process leads to an increase of circulating non-esterified fatty acids (NEFA) such as β-hydroxybutyrate (BHB) after a certain delay to an enlargement of ketone body concentration when hepatic NEFA utilization is insufficient [4,5]. Spreading of ketone bodies involves the risk of ketosis when exceeding critical values. Nielen et al. [6] defined BHB-values in blood serum >1.2 mM as indicator for subclinical ketosis. According to Oetzel [7] NEFA-values >0.4 mM describe a stage were higher lipid mobilization takes place and, therefore, indicate an imbalance in the energy status.

Experimental determination of adipose tissue mobilization usually requires expensive comparative slaughter techniques, which limits the investigation of kinetics of mobilization of individual cows. Using the slaughter technique, Von Soosten et al. [8] determined a mean mobilization of 20 kg during the first 42 days (d) after parturition in heifers. Drackley et al. [9] measured values of 26.5–48.3 kg for mobilization of visceral adipose tissues and 31.8–57.2 kg for abdominal adipose tissues in dry cows. However, little is known about the quantity of adipose tissue mobilization in high lactating, pluriparous cows during the transition period. Using an ultrasound-based technique which was calibrated with simultaneously slaughter-based fat depots, facilitates tracking the development of fat depot masses and consequently of their mobilization and accretion on an individual basis [10]. Thus, this technique was used in the present study to characterize the dynamics of adipose tissue metabolism of transit cows.

Furthermore, the literature reveals a discrepancy about whether a higher body condition increases the body fat mobilization in ruminants [11,12] or not [13]. The same applies for the concentrate proportion and therefore the starch and energy content of the ration. Studies differ in their results when high energy levels of the diet decrease the mobilization of adipose tissues [12,14] or not [13,15]. Hence, a second aim of the experiment was to characterize the deflections in the dynamics of adipose tissue around parturition along with milking performance and parameters of energy metabolism as triggered by varying concentrate feed proportions and different body condition.

## 2. Materials and Methods

The experiment was performed in compliance with the German legislation on animal protection (Animal Welfare Act) and approved by the Lower Saxony State Office for Consumer Protection and Food Safety (LAVES, Oldenburg, Germany) in consultation with an independent ethics committee (AZ 33.19-42502-04-15/1858).

### 2.1. Experimental Design

Sixty pluriparous German Holstein cows were involved in the study from 42 d antepartum (a.p.) relative to calculated calving until 120 d postpartum (p.p.). The experimental design was a 2 × 2 factorial layout with body condition score (BCS) and concentrate proportion in the diet (C) as factors. Cows were either in a high or a low BCS group (BCS_H_ or BCS_L_). Further criteria for allocating the animals were milk yield and milk composition of the previous lactation as well as body weight and number of lactation. Supply of energy and nutrients was ensured based on the recommendations of the Society of Nutrition Physiology [16]. Before parturition, both groups received the same total mixed ration (TMR) consisting of 80% silage (70% maize silage, 30% grass silage on dry matter DM basis) and 20% concentrate on a DM basis. After parturition, the diet changed to two partial mixed rations (PMR) consisting of 48% maize silage, 20% grass silage and 32% concentrate. To subdivide the BCS groups, diets varied in C. Rations for the groups with lower concentrate proportion (C_35_) contained 35% concentrate and an energy content of 6.9 MJ NE_L_/kg DM. The lower C was chosen to stimulate lipolysis by means of an energetic undersupply [4]. For the groups with a higher amount of concentrate (C_60_), C increased from 35–60% during the first three weeks p.p. The C_60_ ration contained an energy content of 7.3 MJ NE_L_/kg DM. Additional concentrate was provided by automatic feeding stations (Insentec, B.V., Marknesse, The Netherlands) until the required amounts were achieved. All cows remained in treatment until day (d) 120 in milk (DIM). Cows in the BCS_H_ group started with an average BCS of 3.83 (±0.41 standard deviation SD). The BCS_L_ group had an average BCS of 3.1 (±0.38 SD). Cows of group BCS_H_/C_60_ (n = 15) had an average parity of 3.4 (±1.1 SD). For the BCS_H_/C_35_ (n = 15) group the average parity was 3.3 (±1.2 SD). Cows of the BCS_L_/C_60_ (n = 15) group had an average parity of 2.6 (±0.9 SD) and for the BCS_L_/C_35_ (n = 15) group the average parity was 2.5 (±0.6 SD). TMR and PMR were provided ad libitum by self-feeding stations (RIC, Insentec B.V., Marknese, The Netherlands). The components and the chemical compositions of the feedstuffs are presented in Table 1 and Table 2.

### 2.2. Sample and Data Collection

Samples of the mixed rations components were taken twice weekly and then pooled to a collective sample for periods of 4 weeks. Samples of concentrate were collected once a week and pooled to a collective sample every 4 weeks, as well. Through the whole experiment, dry matter intake (DMI) was recorded individually for both PMR and concentrate provided by computerized feeding stations. Live weight was recorded weekly a.p. and twice daily p.p. after each milking. Milk yield was quantified twice daily during milking at 0530 and 1530 h by automatic milk counters (Lemmer Fullwood GmbH, Lohmar, Germany). Milk samples were taken twice a week during morning and evening milking and stored at 4 °C until analysis. BCS was determined at noon in weekly intervals on a 5-point-scale according to Edmonson et al. [17].

Blood samples were taken at determined time points, whereby deviations of 2 d were tolerated (42 d a.p., 14 d a.p., 7 d a.p., 3 d a.p., 3 d p.p., 7 d p.p., 14 d p.p., 21 d p.p., 28 d p.p., 42 d p.p., 56 d p.p., 70 d p.p. 95 d p.p. and 120 d p.p.) from the vena jugularis externa. Blood samples were centrifuged (Heraeus Varifuge 3.0R Heraeus, Osterode, Germany; 2123× *g*, 15 °C, 15 min) and stored at −80 °C until further analysis.

Ultrasonic measurements (USM) of adipose tissues took place at defined points in time, too (d 3 p.p., d 28 p.p., d 70 p.p. and d 120 p.p.). Back fat thickness (BFT) was estimated according to Staufenbiel [18], while rib fat thickness (RFT), subcutaneous (SAT), retroperitoneal (RAT), mesenteric (MAT) and omental (OAT) adipose tissues were assessed according to Raschka et al. [10]. For this purpose, double measurement of each tissue were performed using a Mindray M5 Vet (Mindray, Shenzhen, China) diagnostic ultrasound system with a linear (g MHz, Mindray 6LE5Vs) and a convex probe (3 MHz, Mindray 3C5s). The thickness of the measured fat tissues was sized in millimeters. To calculate the adipose tissue depot masses in kg the measuring points established by Raschka et al. [10] were used (Table A1). For calculating the mobilization of adipose tissue depots, the experiment was divided in periods (period 1: weeks 1–4 postpartum, period 2: weeks 5–10 postpartum, period 3: weeks 11–17 postpartum), which were also used for analyzing the remaining parameters to receive a consistent statistical design.

One animal could not be evaluated due to erroneous, implausible values.

### 2.3. Analyses

PMR components and concentrate were analyzed for DM, crude ash, crude protein, ether extract, crude fiber, neutral detergent fiber (NDF_om_) and acid detergent fiber (ADF_om_) according to the standard methods of the Association of German Agricultural Analysis and Research Centres [19] (Table 2). Milk samples were analyzed for fat, protein and lactose by an infrared milk analyzer (Milkoscan FT 6000; Foss Electric, Hillerød, Denmark). Using an automatic photometric measurement system (Eurolyser, Type VET CCA, Salzburg, Austria) serum samples were analyzed for β-hydroxybutyrat (BHB), non-esterified fatty acids (NEFA), triglycerides and glucose. According to the classification by Nielen et al. [6] BHB-values in blood serum >1.2 mM were used as indicator for subclinical ketosis.

### 2.4. Calculations

Weekly means of DMI, net energy intake (NEI), net energy balance (NEB), milk yield, and milk components were used for further calculations. Computations of net energy requirements for maintenance (NE_M_) and lactation (NE_L_) as well as for milk energy concentration were based on equations published by the Society of Nutrition Physiology [16]:

NE_M_ (MJ of NE_L_/d) = 0.294 × BW^0.75^ (1)

NE_L_ (MJ of NE_L_/d) = [milk energy concentration (MJ of NE_L_/d) + 0.086] × milk yield (kg/d) (2)

Milk energy (MJ/kg) = 0.38 × milk fat (%) + 0.21 × milk protein (%) + 0.95(3)

The equation published by Gaines [20] was used to calculate the fat corrected milk (FCM):

4% FCM (kg/d) = {[milk fat (%) × 0.15] + 0.4} × milk yield (kg/d)(4)

Energy corrected milk (ECM) was calculated based on the equation by Sjaunja et al. [21]:

ECM (kg/d) = milk yield (kg/d) × {[38.3 × milk fat (g/kg) + 24.2 × milk protein (g/kg) + 16.54 × milk lactose (g/kg) + 20.7]/3140}(5)

In order to calculate the energy intake per day, the energy content of the feedstuffs was multiplied by DMI.

To calculate the NEB, the following equation was used:

NEB (MJ of NE_L_/d) = NEI (MJ of NE_L_/d) − NE_M_ (MJ of NE_L_/d) − NE_L_ (MJ of NE_L_/d) (6)

To regard the gestational requirements in the NEB 13 MJ of NE_L_/d were subtracted from week 6-3 a.p. During the last 3 weeks until calving the requirements were assessed with 18 MJ of NE_L_/d.

For determination of the different adipose tissue depot masses, equations published by Raschka et al. [10] were used based on the collected data of the ultrasonic measurements:

Subcutaneous adipose tissue (SAT, kg) = −6.66 + 0.72 × R12 + 0.31 × AW3c(7)

Retroperitoneal adipose tissue (RAT, kg) = −9.55 + 0.62 × R12 + 0.06 × KD3b(8)

Omental adipose tissue (OAT, kg) = −2.32 + 0.55 × BFT + 0.37 × AW3b(9)

Mesenteric adipose tissue (MAT, kg) = −12.8 + 0.38 × AW1b + 1.73 × AW3b − 1.45 × AW3c + 0.07 × KD2c(10)

Sum of mobilized adipose tissues (SoM, kg) = SAT + RAT + OAT + MAT(11)

Time-dependent changes in the individual and total adipose tissue depots were calculated by the differences of the fat masses between different time points.

It is assumed that 1 g of body fat corresponds to 39.8 kJ gross energy [22] of which 16% is lost as heat when fat is mobilized [23]. The following equation was used to estimate the energy mobilized from the adipose tissue depots:

Mobilized energy = Mobilized fat (kg) × 39.8 MJ/kg × 0.84(12)

We used the following equations according to Hurley et al. [24] to calculate the efficiency parameters feed efficiency (FE), energy conversion efficiency (ECE), metabolic efficiency (MEff) and residual energy intake (REI):

FE = ECM/DMI (kg/kg)(13)

ECE = Energy excretion with milk (MJ)/Energy intake (MJ NE_L_) (14)

MEff = [Energy intake (MJ NE_L_) − Energy excretion with milk (MJ)]/Body weight^0.75^ (kg) (15)

REI = Energy intake (MJ NE_L_) − expected energy intake (MJ NE_L_) (16)

Variables of each of the three periods were finally summarized according to the mobilization of the adipose tissue depots, the experimental weeks of the performance, the milk and blood parameters, as well as the results of the calculated efficiency.

### 2.5. Statistical Analyses

As animals were fed similar diets before parturition, we evaluated the data for the postpartum period only.

The statistical analyses were performed by utilizing the statistical software SAS (version 9.4; SAS Institute Inc., Cary, NC, USA). Performance parameters, blood values, mobilization of adipose tissue depot masses and efficiency parameters were analyzed by using the MIXED procedure for repeated measures with a compound symmetry structure [25]. BCS classification (BCS_H_, BCS_L_), C (C_35_, C_60_) and period (1, 2, 3) were applied as fixed effects, as well as the interactions between them. Each cow within treatment was considered a random effect. The period of sampling was regarded to be a repeated measure. Milk parameters were analyzed with the first measured value in week 1 as covariate. For the remaining parameters, the first measured value before calving was used as covariate. *p*-values ≤ 0.05 were declared to be statistically significant and *p*-values ≤ 0.01 considered highly significant. For calculating correlations between parameters, we employed the statistical software TIBCO Statistica (Version 13.3, TIBCO Software Inc., Palo Alto, CA, USA) by using Pearson’s correlation. The correlation coefficient (r) was considered statistically significant, when *p* ≤ 0.05, and highly significant, when *p* ≤ 0.01. In the following, results are presented as LSMean ± Standard error of means (SEM) unless otherwise stated.

## 3. Results

### 3.1. Performance Parameters

**Figure 1:**
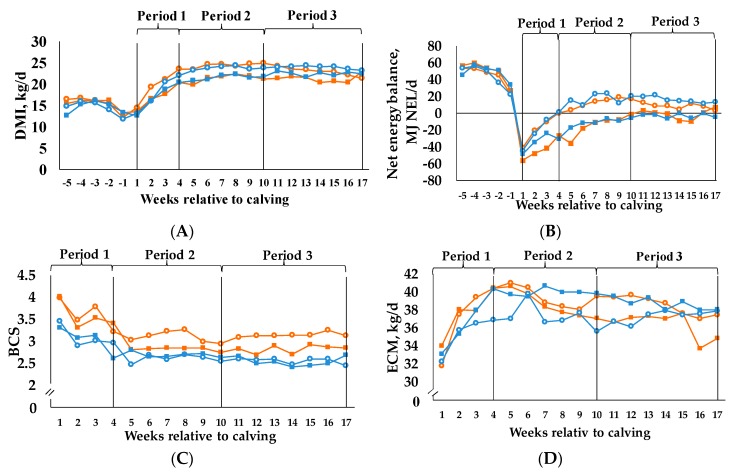
Development of (**A**) dry matter intake (DMI), (**B**) net energy balance, (**C**) body condition score (BCS) and (**D**) energy corrected milk yield (ECM) in the course of the experiment. Cows were categorized in high BCS (BCS_H_) and low BCS (BCS_L_). After parturition, these two groups were divided again, each into a group with a concentrate proportion of 60% (C_60_) in the ration (increasing from 35–60% during the first three weeks after calving) and a group with a concentrate proportion of 35% (C_35_) in the ration. Thus, four groups emerged: BCS_H_/C_60_ (n = 15; **○**), BCS_H_/C_35_ (n = 15; ■), BCS_L_/C_60_ (n = 15; **○**), BCS_L_/C_35_ (n = 15; ■), BCS, DMI and NEB were analyzed with first measured value before calving as covariate, ECM was analyzed with first measured value from week 1 as covariate, (**A**) *p*-values: BCS = 0.761, C = 0.069, period < 0.001, BCS × C = 0.836, BCS × period = 0.232, C × period = 0.042, BCS × C × period = 0.295; (**B**) *p*-values: BCS = 0.540, C < 0.001, period < 0.001, BCS × C = 0.985, BCS × period = 0.639, C × period < 0.001, BCS × C × period = 0.020; (**C**) *p*-values: BCS = 0.004, C = 0.252, period < 0.001, BCS × C = 0.998, BCS × period = 0.011. C × period = 0.182, BCS × C × period = 0.030; (**D**) *p*-values: BCS = 0.679, C = 0.579, period < 0.001, BCS × C = 0.217, BCS × period = 0.003, C × period = 0.043, BCS × C × period = 0.150.

For DMI (Figure 1A) we observed a C × period interaction (*p* = 0.042). The C_35_ groups exhibited a lower DMI than the C_60_ groups over time. All groups had a lower DMI in the first period compared to the following.

The same is true for NEI (Table 3), where we also detected a C × period interaction (*p* = 0.001). C_35_ groups had a significantly lower NEI than C_60_ groups in periods 2 and 3. For NEB (Figure 1B) we observed a treatment x period interaction (*p* = 0.020) as it was more negative in BCS_H_/C_35_ group than in BCS_H_/C_60_ and BCS_L_/C_60_ groups in period 1. Furthermore, NEB was in a positive range for the BCS_H_/C_60_ and BCS_L_/C_60_ groups, but in a negative range for the BCS_H_/C_35_ and BCS_L_/C_35_ groups in period 2. The development of the NEB outlines that the C_60_ groups reached a balanced NEB after four weeks p.p. and remained relatively stable then, whereas the C_35_ groups increased continuously and reached the settlement only at the end of the experiment.

For BCS (Figure 1C) we proved a treatment x period interaction (*p* = 0.030). The BCS_H_/C_60_ differed from the BCS_L_/C_60_ and BCS_L_/C_35_ group in all three periods, due to higher mean values. Moreover, the BCS_H_/C_35_ showed higher means in comparison to the BCS_L_/C_35_ group in period 1.

Live weight (Table 3) decreased from period 1 to 2, whereas it increased from period 2 to 3 within the C_35_ and C_60_ groups. We found a C × period interaction (*p* = 0.018), but no differences within one period.

### 3.2. Milk Parameters

For milk yield (Table 4 and Table 5) we detected two interactions, BCS × period (*p* = 0.023) and C × period (*p* < 0.001). However, neither BCS nor C had an influence within the same period. For milk fat content (Table 4 and Table 5) the same interactions were determined (BCS × period: *p* < 0.001, C × period: *p* = 0.001). The higher BCS led to a higher milk fat content in period 1, the same did a lower C in periods 2 and 3.

This is similar to the C × period interaction (*p* = 0.001) for milk fat yield (Table 4 and Table 5), where lower C also led to higher means in period 2. For milk protein content (Table 4 and Table 5), we found a C × period interaction (*p* = 0.021), too. There were no differences within the same period. Period (*p* < 0.001) had an effect on milk protein yield (Table 4 and Table 5), as period 1 differed from periods 2 and 3 due to higher milk protein yields for the latter two periods in all four groups. We discovered a BCS × C × period interaction (*p* = 0.013) for milk lactose content (Table 4 and Table 5). In period 1 the BCS_L_/C_60_ group differed from the BCS_L_/C_35_ group with regard to a higher mean. The same interaction was determined for milk lactose yield (BCS × C × period: *p* = 0.005, Table 4 and Table 5), although the groups exhibited no differences within the same period. We observed a C × period interaction for milk fat:protein ratio (Table 4 and Table 5), where lower C again led to higher values in all three periods. For milk energy concentration (Table 4 and Table 5), we observed a BCS × C × period interaction (*p* = 0.017). In periods 2 and 3 the BCS_H_/C_35_ group differed from the BCS_L_/C_60_ group regarding a higher energy concentration. The BCS_L_/C_60_ group and the BCS_L_/C_35_ group exhibited different means in period 3 only, whereby the former showed a higher energy concentration. We determined a BCS × period interaction (*p* = 0.003) and a C × period interaction (*p* = 0.040) for milk energy output (Table 4 and Table 5). However, no differences within the same periods were found. The same is true for the 4% FCM (Table 4 and Table 5). We determined the same interactions (BCS × period: *p* = 0.008, C × period: *p* = 0.044), but did not observe any differences within same periods. We found those two interactions (BCS × period: *p* = 0.003, C × period: *p* = 0.040) again for ECM (Figure 1D), but once more there were no differences within the same periods quantifiable.

### 3.3. Mobilization of Adipose Tissue Depots and Energy

BCS influenced BFT (Table 6) over time as we found a BCS × period interaction (*p* = 0.005). However, we could not determine differences between groups within one period. For RFT (Table 6) a time effect (*p* < 0.001) was observed. In all groups RFT was more degraded in period 1 than in periods 2 and 3.

**Figure 2:**
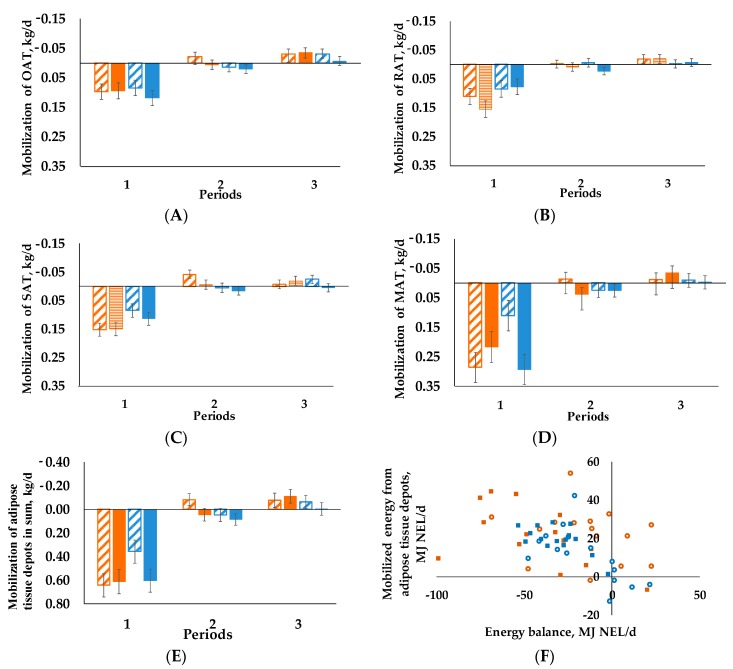
Mobilization of the single adipose tissues and the sum of the (**A**) omental (OAT), (**B**) retroperitoneal (RAT), (**C**) subcutaneous (SAT), (**D**) mesenteric (MAT), (**E**) adipose tissues, negative values represent accretion of adipose tissue, positive values describe mobilization, LSMeans) of the experimental groups during the three periods, period 1: weeks 1–4 postpartum, period 2: weeks 5–10 postpartum, period 3: weeks 11–17 postpartum); as well as the (**F**) correlation of mobilized energy from adipose tissues and energy balance for each individual cow in period 1 (correlation coefficient = −0.4634, *p* < 0.05). Cows were categorized in high BCS (BCS_H_) and low BCS (BCS_L_). After parturition, these two groups were divided again, each into a group with a concentrate proportion of 60% (C_60_) in the ration (increasing from 35–60% during the first three weeks after calving) and a group with a concentrate proportion of 35% (C_35_) in the ration. Thus, four groups emerged: BCS_H_/C_60_ (n = 15; **A**–**E** orange striped bars, **F** **○**) BCS_H_/C_35_ (n = 14; **A**–**E** orange bars, **F** ■), BCS_L_/C_60_ (n = 15; **A**–**E** blue striped bars, **F** ○), BCS_L_/C_35_ (n = 15; **A**–**E** blue bars, **F** ■). Error bars indicate SEM. Mixed models are analyzed with first measured value before calving as covariate, (**A**) *p*-values: BCS = 0.216, C = 0.213, period < 0.001, BCS × C = 0.536, BCS × period = 0.734, C × period = 0.953, BCS × C × period = 0.382; (**B**) *p*-values: BCS = 0.428, C = 0.381, period < 0.001, BCS × C = 0.648, BCS × period = 0.089, C × period = 0.526, BCS × C × period = 0.382; (**C**) *p*-values: BCS = 0.740, C = 0.250, period < 0.001, BCS × C = 0.549, BCS × period = 0.002, C × period = 0.788, BCS × C × period = 0.244; (**D**) *p*-values: BCS = 0.703, C = 0.163, period < 0.001, BCS × C = 0.034, BCS × period = 0.480, C × period = 0.366, BCS × C × period = 0.065; (**E**) *p*-values: BCS = 0.984, C = 0.138, period < 0.001, BCS × C = 0.311, BCS × period = 0.087, C × period = 0.595, BCS × C × period = 0.232.

The same effect is true for OAT (Figure 2A, *p*_period_ < 0.001) and RAT (Figure 2B, *p*_period_ < 0.001). In contrast, BCS had an influence over time concerning SAT (Figure 2C), as we detected a BCS × period interaction (*p* = 0.002). In period 1 we observed higher mobilization for BCS_H_/C_60_ and BCS_H_/C_35_ than for BCS_L_/C_60_ and BCS_L_/C_35_. Apart from that we found a BCS × C interaction (*p* = 0.028) for MAT (Figure 2D).

Neither C nor BCS had an influence on SoM (Figure 2E), only a time effect (*p*_period_ < 0.001) was visible. Period 1 differed from periods 2 and 3 concerning higher mobilization in all groups. For period 2 the BCS_H_/C_60_ group exhibited accretion, which is also true for all groups in period 3.

### 3.4. Blood Parameters

**Figure 3:**
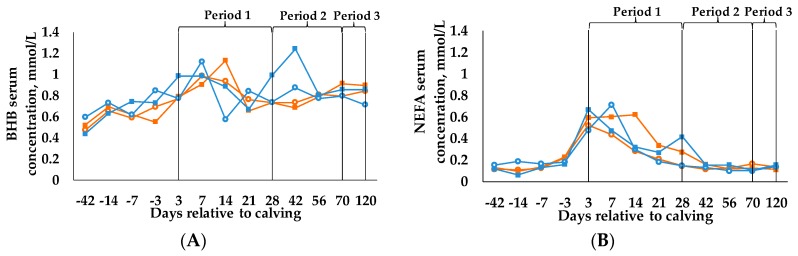
Concentration of (**A**) β-hydroxybutyrat (BHB) and (**B**) non-esterified fatty acids (NEFA) in blood serum (LSM) from d 42 antepartum until d 120 postpartum. Statistical analysis starts at calving (period 1: weeks 1–4 postpartum, period 2: weeks 5–10 postpartum, period 3: weeks 11–17 postpartum). Cows were categorized in high BCS (BCS_H_) and low BCS (BCS_L_). After parturition, these two groups were divided again, each into a group with a concentrate proportion of 60% (C_60_) in the ration (increasing from 35–60% during the first three weeks after calving) and a group with a concentrate proportion of 35% (C_35_) in the ration. Thus, four groups emerged: BCS_H_/C_60_ (n = 15; **○**), BCS_H_/C_35_ (n = 15; ■), BCS_L_/C_60_ (n = 15; **○**), BCS_L_/C_35_ (n = 15; ■), Parameters are analyzed with first measured value before calving as covariate, (**A**) *p*-values: BCS = 0.747, C = 0.346, period = 0.906, BCS × C = 0.621, BCS × period = 0.272, C × period = 0.762, BCS × C × period = 0.422; (**B**) *p*-values: BCS = 0.877, C = 0.208, period < 0.001, BCS × C = 0.909, BCS × period = 0.557, C × period = 0.131, BCS × C × period = 0.069.

Results for BHB (Figure 3A) and NFEA (Figure 3B) are presented in d, as the measuring points were determined in d relative to calving. Nevertheless, in accordance with the other data, the statistical analyses are calculated in periods and start at calving.

Neither BCS and C, nor time affected the concentration of BHB in blood serum. Only the BCS_L_/C_35_ group exceeded the threshold of 1.2 mmol/L declared as an indicator for subclinical ketosis according to Nielen et al. [6] in period 2.

For NEFA, we observed a time effect (*p* < 0.001) as concentration in blood serum was highest in period 1 and decreased in periods 2 and 3.

The same is true for glucose (Table A2) as a time effect (*p* < 0.001) was determined. Like in the case of NEFA, the concentration of glucose in blood serum was lower in period 1 than in periods 2 and 3, regardless of BCS and C.

Triglycerides (Table A2) were influenced by C, as C_60_ groups presented higher values than C_35_ groups. Furthermore, triglycerides exhibited a BCS × period interaction (*p* = 0.034). However, there were no differences within the same period. In accordance to the preceding parameter, the concentration of triglycerides was lowest in period 1 in all experimental groups.

### 3.5. Efficiency Parameters

To compare the different energy and feed efficiencies between groups, efficiency variables were calculated. The C affected FE (Table A3 and Table A4), as we observed a C effect (*p* = 0.003) as well as a time effect (*p* = 0.001). Groups with a lower concentrate proportion were more efficient. In period 1 all groups showed the highest FE compared to periods 2 and 3. The same is true for ECE (Table A3 and Table A4) were we determined the same effects *(p*_C_ < 0.001, *p*_period_ < 0.001). Again, groups with lower concentrate proportion had higher ECE values because of a higher efficiency. In accordance with the FE, ECE values were higher in period 1 than in periods 2 and 3 in all groups.

MEff (Table A3 and Table A4) exhibited a C × period interaction (*p* < 0.001). Groups with a lower concentrate proportion in the ration had lower means in all three periods.

We found the same results for REI (Table A3 and Table A4). Similarly, to MEff, groups with lower concentrate availability had lower means during the whole trial.

### 3.6. Correlations

Relations of elevated parameters were computed using correlations. The following values were considered: total mobilization of adipose tissue depots both in sum and separately as well as NEB, BHB, NEFA and efficiency parameter FE.

For all animals NEB correlated negatively with BFT(r = −0.466, *p* ≤ 0.001), RAT (r = −0.349, *p* ≤ 0.01), SAT (r = −0.397, *p* ≤ 0.01), MAT (r = −0.271, *p* ≤ 0.05) and SoM (r = −0.375, *p* ≤ 0.01), as well as with BHB (r = −0.502, *p* ≤ 0.001), NEFA (r = −0.402, *p* ≤ 0.01) and FE (r = −0.867, *p* ≤ 0.001). By contrast BCS, RFT and OAT did not correlate. The mobilized energy from adipose tissue depots and NEB (Figure 2F) showed a significant negative correlation, as correlation coefficient was −0.4634 (*p* < 0.05) for period 1.

## 4. Discussion

The aim of this study was to investigate the body fat depot mobilization and energy metabolism of pluriparous cows during the first weeks after parturition depending on body condition before calving and on different amounts of concentrate in the ration after calving by combining a feeding trial with ultrasound-based estimation of various depot fat depots.

One outcome of our investigation was that the amount of concentrate in the ration influenced the DMI, as groups with higher supply of concentrate consumed more DM in period 2. This result is comparable to other studies. Schmitz et al. [26] showed that a high proportion of concentrates in the ration enhanced DMI and Gruber et al. [27] proposed an increase between 0.4–0.6 kg per kg additional concentrate, which was linked to a subsequent roughage displacement. In contrast, other findings pointed out that there were no differences in feed intake between cows fed rations with different proportions of concentrates during the lactation period [28]. A reason for these controversial results might be caused by other feeding factors, such as composition, energy density and quality of roughage, as well as NDF content.

The higher DMI of C_60_ groups of our trial also resulted in a higher energy intake compared to C_35_ groups. These relations might explain why low concentrate groups suffered from a more pronounced negative EB and reached the positive EB much later than high concentrate groups. As described in Dänicke et al. [29] the energetic dilution of the C_35_ ration leads to a qualitative decrease of NEB.

Differences in BCS between both BCS groups and between individuals remained more or less the same over the whole transition period, whilst higher concentrate feed supply after calving (C_60_) failed to counterbalance the BCS loss observed in group with lower concentrate allowance (C_35_). Other studies agree with our findings, that feeding has little effect on BCS loss after parturition [9,30]. As NEI was higher in group C_60_ and NEB less negative, the question was whether the mobilization of the internal fat depots was less pronounced. However, the individual groups of our trial did not differ when SoM was evaluated collectively. Having a closer look at individual fat depots, it becomes obvious that SAT was more extensively mobilized in BCS_H_ groups compared to their BCS_L_ counterparts. This finding is surprising as no corresponding differences in BCS losses were noticed and BCS changes are usually regarded to represent the changes in SAT [31]. This significant effect of BCS on SAT mobilization exclusively occurred in period 1 where mobilization of all measured fat depots was most pronounced. The metabolic circumstances and the pronounced negative EB during this time explain these expectable results, which are also proved by the negative correlation of mobilized energy from body fat and NEB. Tamminga et al. [3] share our findings. With regard to the effect of body condition on mobilization of SAT Chilliard et al. [11] have shown that fat mobilization was physiologically related to body fatness. Nevertheless, we could not demonstrate the expected correlation between BCS and NEB, which would have indicated the higher potential of high-conditioned cows to mobilize body fat. This non-existent relationship could also explain why we determined no further association between BCS and mobilization. That is in line with Pedernera et al. [14] who had already pointed out, that BCS should not be used as an overall parameter to explain the energetic condition and metabolism of cows. It becomes apparent that USM show differences in lipid mobilization, which remain concealed by simply determining the BCS.

Due to the limited capability of BCS changes to indicate fat mobilization comprehensively, one could hypothesize that the mobilization of adipose tissues was related to energy balance, as proven by the highly significant overall correlations between NEB and mobilized energy from body fat, as well as those of NEB and the single adipose tissue depots and SoM in all animals of our investigation. Consequently, it should also depend on C whereby a decline of the energy balance would expectedly increase the lipolytic potential [11]. Reversely, high amounts of concentrate would then lead to a positive EB and consequently result in a decrease of adipose tissue mobilization. Our study could, however, not support those findings and other trials, which attempted to decrease body fat mobilization by increasing energy-rich diets, had neither been successful [15]. Considering the NEB alone does not duly reflect the metabolic processes. This suggests that other factors, such as genetic regulatory mechanisms may influence the mobilization relevantly.

Blood concentration of BHB is used as an indicator for lipolysis and ketosis whereby the literature states different thresholds. Nielen et al. [6] defined a value of BHB > 1.2 mmol/L in serum as the critical level for subclinical ketosis. Oetzel [7] declared a level of NEFA > 0.4 mmol/L as a stage where high lipomobilization takes place and therefore indicates an imbalance in energy state.

In the present study, none of the four trial groups exceeded the limit value for BHB in period 1, and we could not demonstrate any time or group effects, too. NEFA values were not affected by treatment either, but showed a significant time effect and also the characteristic curve during the transition period. NEFA values are typically higher in early lactation than in mid-lactation [32] which indicates higher mobilization of adipose tissue depots due to the particular metabolic challenges during this time. All four groups of our study exceeded the threshold for higher lipomobilization (>0.4 mmol/L) in period 1 [7]. This is in line with the negative energy balance during this timeframe as this threshold indicates a negative energy state. The negative EB was more pronounced in groups with lower concentrate allowance; due to a forced lipid mobilization, we would have also expected higher serum concentrations for both BHB and NEFA. However, the sonography-based determination of the adipose tissue mobilization did not verify the differences seen in NEB. A closer look at SAT, MAT and SoM indicated that regular conditioned animals with a lower concentrate proportion in the ration mobilized as much body fat as regular conditioned cows with a high concentrate availability. Only cows with an energetic oversupply mobilize less body fat. Van der Drift [33] stated that a high BCS and higher fat depot mobilization increased the risk for ketosis and hypothesized that high-energy diets compensated the cows’ energy demand and made high mobilization of body fat unnecessary in order to avoid that. Other studies confirm this presumption [12,14,34]. Chilliard [35] pointed out that cows mobilize more body fat when their access to feed is limited. Other studies argue the converse. Cows with high-energy diets exhibited higher BHB and NEFA values compared with cows supplied with low-energy diets [4,34]. Yet other studies, in turn, go in line with our findings and could not determine any significant differences between treatments in the first weeks p.p. [26,36]. This suggests that NEB does not accurately reflect the grade of lipid mobilization. The examination of lipid mobilization by ultrasonic technology brings out physiological and metabolic relations that remain concealed by using BCS determination or NEB calculation only.

A possible explanation for our results might be the absence of lipolytic stimuli in the adipose tissue depots, for example, a low glucose level [37]. Propionate is necessary for synthesizing glucose. Presumably, in the present study, there might have been sufficient DMI and also starch and energy content in both rations to generate adequate amounts of propionate in the rumen, so that glucose concentration did not decrease and therefore no lipolytic stimuli occurred. Another reason for detecting only concentrate tendencies and a single BCS effect on fat depot mobilization could be an adequate level of oxaloacetate for introducing NEFA into the citric acid cycle which prevents an increase of BHB beyond the physiological range [38]. Van der Drift [33] indicates that not only non-genetic, but also genetic variations of BHB concentration have to be considered. Those findings reflect our perceptions concerning SoM, where we could not prove any group differences either. Different studies had already pointed out that cows vary in their potential to deal with metabolic changes and to adapt to NEB and varying dietary energy [26,33,39].

In the present study, the mobilization of protein was not examined. Protein mobilization can reduce both, fat mobilization and NEFA and BHB concentration in blood [33]. It can be hypothesized that C_35_ and BCS_H_ groups mobilized sufficient protein to cover group effects in fat mobilization and serum NEFA and BHB concentration.

A positive relationship between C and milk yield would have been expected due to the additionally provided energy in the diet as had been proved in previous studies [12,26,40]. BCS had no influence on milk yield either. Other investigations underline our findings that body condition at calving has little effect on milk production of well-fed cows [35]. In the present study we failed to demonstrate a BCS effect both on DMI and on milk yield. Nevertheless, other milk parameters such as milk fat content differed between trial groups in the present study. The higher BCS might have increased the milk fat content caused by higher mobilization of body fat reserves in period 1 [12,34]. Furthermore, higher amounts of concentrates led to milk fat depression in periods 2 and 3, because of the associated decrease of the acetate-to-propionate ratio in the rumen [41,42]. Acetate is an important source for synthesis of milk fat in ruminants [43]. Lipid mobilization of BCS_H_ groups may have concealed the concentrate effect in period 1. These results are also reflected in ECM. Relating to NEB, lower ECM yield and higher DMI led to a positive EB in C_60_ groups.

In our trial, the C_35_ groups seem to be more efficient, as their FE was higher compared to C_60_ groups. This is, however, related to an equal milk production level of both groups, whereby animals of C_35_ groups consumed less DMI containing the lower energy content. Thus, C_35_ groups also exhibited a longer and more pronounced negative energy balance. In this case, higher milk production was accompanied by less feed intake and more body fat mobilization. Spurlock et al. [44] proposed a genetic correlation between high FE and a more pronounced negative energy balance. We could underline this due to the highly significant correlation between NEB and FE in the present study. It indicates that efficient cows might be more endangered to develop metabolic diseases. This hypothesis is confirmed by findings of Chilliard et al. [11], where NEFA concentrations were highly correlated with NEB, which could also be proven in the present study.

## 5. Conclusions

The results of this study confirm the benefits of higher amounts of concentrates concerning the DMI, and therefore the energy intake, whereas milk yield was unaffected. However, higher amounts of concentrate led to milk fat depression. Due to higher energy intake and equal milk yield, C_60_ groups reached the positive energy balance earlier than the C_35_ groups. Lower DMI and equal milk yield led to an improved efficiency in C_35_ groups. However, the term efficiency must be critically reviewed, as it is related to a high energy deficit.

The determination of body fat mobilization by ultrasonic recording revealed the differences between groups seen in the NEB, which suggested a need for higher lipid mobilization in C_35_ groups.

Furthermore, we could not find differences between groups concerning BCS loss. However, BCS affected the mobilization of SAT over time, as high-conditioned cows had a more pronounced SAT mobilization. Although BCS is based on changes in SAT, the determination failed to detect them in the present study. Therefore, BCS determination and NEB calculation should be used carefully as indicators for general mobilization or overall metabolic processes and conditions. The sonography-based method revealed boundaries of BCS determination and NEB calculation and visualized physiological and metabolic relations that remain concealed in other methods. Physiological processes, such as protein mobilization and hormone levels, might also influence fat loss. Further research is necessary to clarify causal interrelations.

## Figures and Tables

**Table 1:** Composition of concentrates during the dry and the lactating periods.

Components, g/kg of Fresh Matter	Concentrates		
	Dry Period	C_35_	C_60_
Soybean meal	115		
Rapeseed meal	150	400	200
Wheat	330	150	213
Barley		144	213
Maize		200	290
Dried sugar beet pulp	296	50	50
Urea	30	8	
Calcium carbonate	24	13	12
Soybean oil	15	10	10
Vitamin-mineral premix ^+^	40		
Vitamin-mineral premix ^#^		25	12

^+^ Mineral feed for dry cows, ingredients per kg according to the manufacturer’s specification: 10 g Ca; 120 g Na; 60 g P; 60 g Mg; 6 g Zn; 4 g Mn; 1.25 g Cu; 100 mg I; 50 mg Se; 35 mg Co; 8,000,000 IU vitamin A; 1,000,000 vitamin D_3_; 2500 mg vitamin E, ^#^ Mineral feed for lactating dairy cows, ingredients per kg according to the manufacturer’s specifications: 140 g Ca; 120 g Na; 70 g P; 40 g Mg; 6 g Zn; 5.4 g Mn; 1 g Cu, 100 mg I; 40 mg Se; 25 mg Co; 1,000,000 IU vitamin A; 1,000,000 IU vitamin D_3_; 1500 mg vitamin E.

**Table 2:** Chemical components of concentrates and roughage during the experimental period from day 42 antepartum until day 120 postpartum.

Chemical Composition	Concentrates			Roughage	
	Dry Period	C_35_ *	C_60_^#^	Maize Silage	Grass Silage
Dry matter, g/kg	890	878	878	361	306
Nutrients, g/kg DM ^§^					
Crude ash	90	76	55	40	94
Crude protein	277	239	170	82	127
Ether extract	42	48	48	32	33
Crude fiber	94	85	66	203	273
a^†^Neutral detergent fiber_om_^‖^	207	208	178	401	526
Acid detergent fiber_om_^‖^	125	125	96	230	297
Starch content	310	368	490	331	0
Energy ^‡^, MJ/kg of DM					
ME	11.9	12.3	12.8	10.8	10.4
NE_L_	7.4	7.7	8.1	6.5	6.2

^‡^ Calculation based on equations of GfE [16]. ^§^ Dry matter. ^†^ Assayed with a heat-stable amylase. ^‖^ Expressed exclusive of residual ash. * Total starch content of C_35_-ration was 285 g/kg DM. Total energy content of C_35_-ration was 11.3 MJ ME/kg DM and 6.9 MJ NE_L_/kg DM. ^#^ C_60_-ration contained a starch content of 353 g/kg DM. Total energy content of C_60_-ration was 11.8 MJ ME/kg DM and 7.3 MJ NE_L_/kg DM.

**Table 3:** Effects of body condition, concentrate proportion in the diet (C) and period on dry matter intake (DMI), energy intake and live weight (LSM) during period 1 (weeks 1–4 postpartum), period 2 (weeks 5–10 postpartum) and period 3 (weeks 11–17 postpartum) in the treatment groups.

Item ^+^	Treatment ^§^				SEM ^#^	*p*-Value *					
	BCS_H_/C_60_ n = 15	BCS_H_/C_35_ n = 15	BCS_L_/C_60_ n = 15	BCS_L_/C_35_ n = 15		BCS	C	BCS × C	BCS × Period	C × Period	BCS × C × Period
DMI											
Period 1	19.4	17.7	18.6	18.0	0.6	0.761	0.069	0.836	0.232	0.042	0.295
Period 2	24.3	22.6	24.4	22.9							
Period 3	23.4	23.3	24.3	23.6							
Energy intake, MJ of NE_L_/day											
Period 1	143	124	131	128	4	0.949	<0.001	0.252	0.144	0.001	0.069
Period 2	182	157	179	163							
Period 3	175	163	177	166							
Live weight, kg											
Period 1	706	663	701	673	14	0.865	0.069	0.726	0.800	0.018	0.225
Period 2	694	662	688	670							
Period 3	699	677	703	682							

^§^ Before calving cows were classified in high and low body condition score (BCS)-groups (BCS_H_, BCS_L_), after calving both groups were divided again, each into a group with 35% concentrate proportion (C_35_) and a group with 60% concentrate proportion (C_60_) in the ration (increasing from 35–60% during the first three weeks after parturition). Thus, four groups emerged: BCS_H_/C_60_ (n = 15), BCS_H_/C_35_ (n = 15), BCS_L_/C_60_ (n = 15), BCS_L_/C_35_ (n = 15). Values are presented as LSMeans, * Period (*p* < 0.001) for all variables, ^+^ Analyzed with first measured value before calving as covariate, ^#^ Pooled standard error of means.

**Table 4:** Effects of body condition, concentrate proportion in the diet (C) and period on milk parameters (LSM) during period 1 (weeks 1–4 postpartum), period 2 (weeks 5–10 postpartum) and period 3 (weeks 11–17 postpartum) in the treatment groups.

Item ^+^	Treatment ^§^				SEM ^#^
	BCS_H_/C_60_ n = 15	BCS_H_/C_35_ n = 15	BCS_L_/C_60_ n = 15	BCS_L_/C_35_ n = 15	
Milk yield, kg/day					
Period 1	32.8	32.2	32.1	33.8	1.1
Period 2	42.4	38.9	41.8	42.4	
Period 3	41.2	37.6	42.1	40.9	
Milk fat content, %					
Period 1	4.68	4.94	4.33	4.52	0.15
Period 2	3.23	3.79	3.19	3.83	
Period 3	3.26	3.58	3.06	3.73	
Milk fat yield, kg/day					
Period 1	1.62	1.63	1.49	1.61	0.06
Period 2	1.37	1.54	1.30	1.57	
Period 3	1.34	1.41	1.26	1.49	
Milk protein content, %					
Period 1	3.49	3.35	3.39	3.37	0.05
Period 2	3.23	3.18	3.24	3.15	
Period 3	3.30	3.27	3.24	3.27	
Milk protein yield, kg/day					
Period 1	1.21	1.14	1.18	1.20	0.03
Period 2	1.37	1.27	1.34	1.31	
Period 3	1.34	1.26	1.37	1.32	
Milk lactose content, %					
Period 1	4.77 ^c,A^	4.58 ^c,B^	4.72 ^c,AB^	4.75 ^c,AB^	0.03
Period 2	4.88 ^b^	4.85 ^b^	4.80 ^b^	4.83 ^b^	
Period 3	4.91 ^a^	4.90 ^a^	4.87 ^a^	4.85 ^a^	
Milk lactose yield, kg/day					
Period 1	1.67 ^b^	1.60 ^b^	1.64 ^b^	1.71 ^b^	0.05
Period 2	2.07 ^a^	1.88 ^a^	2.01 ^a^	2.01 ^a^	
Period 3	2.01 ^a^	1.89 ^a^	2.06 ^a^	1.96 ^a^	
Milk fat:protein ratio					
Period 1	1.35	1.43	1.25	1.31	0.04
Period 2	1.03	1.23	0.95	1.18	
Period 3	1.02	1.14	0.91	1.11	
Milk energy concentration, MJ/kg					
Period 1	3.65 ^a^	3.74 ^a^	3.48 ^a^	3.51 ^a^	0.06
Period 2	2.98 ^b,AB^	3.23 ^b,A^	2.88 ^b,B^	3.14 ^b,AB^	
Period 3	3.01 ^b,AB^	3.16 ^b,A^	2.84 ^b,B^	3.13 ^b,AB^	
Milk energy output, MJ/day					
Period 1	124.3	125.7	118.1	122.9	3.6
Period 2	131.7	129.4	125.7	133.9	
Period 3	128.0	122.0	125.4	129.3	
4% FCM, kg/day					
Period 1	37.5	38.4	35.3	37.0	1.2
Period 2	38.4	38.8	36.3	39.9	
Period 3	37.3	36.1	36.0	38.1	

^a,b^ Means with different superscripts differ within columns, ^A,B^ Means with different superscripts differ within row, ^§^ Before calving cows were classified in high and low body condition score (BCS)-groups (BCS_H_, BCS_L_), after calving both groups were divided again, each into a group with 35% concentrate proportion (C_35_) and a group with 60% concentrate proportion (C_60_) in the ration (increasing from 35–60% during the first three weeks after parturition). Thus, four groups emerged: BCS_H_/C_60_ (n = 15), BCS_H_/C_35_ (n = 15), BCS_L_/C_60_ (n = 15), BCS_L_/C_35_ (n = 15). Values are presented as LSMeans. ^+^ Analyzed with first measured value from week 1 as covariate, ^#^ Pooled standard error of means.

**Table 5:** *p*-values of effects of body condition, concentrate proportion in the diet (C), period and interactions between them on milk parameters.

Item ^+^	*p*-Value *					
	BCS	C	BCS × C	BCS × Period	C × Period	BCS × C × Period
Milk yield, kg/day	0.205	0.302	0.181	0.026	<0.001	0.229
Milk fat content, %	0.362	0.002	0.655	<0.001	<0.001	0.059
Milk fat yield, kg/day	0.580	0.013	0.296	0.165	0.001	0.738
Milk protein content, %	0.574	0.290	0.631	0.777	0.019	0.091
Milk protein yield, kg/day	0.480	0.069	0.267	0.129	0.083	0.320
Milk lactose content, %	0.642	0.244	0.083	0.010	0.107	0.013
Milk lactose yield, kg/day	0.372	0.187	0.268	0.533	0.006	0.005
Milk fat:protein ratio	0.05	0.001	0.749	0.211	<0.001	0.163
Milk energy concentration, MJ/kg	0.035	0.004	0.881	0.022	<0.001	0.018
Milk energy output, MJ/day	0.780	0.633	0.245	0.003	0.040	0.192
4% FCM, kg/day	0.566	0.278	0.288	0.009	0.046	0.169

Before calving cows were classified in high and low body condition score (BCS)-groups (BCS_H_, BCS_L_), after calving both groups were divided again, each into a group with 35% concentrate proportion (C_35_) and a group with 60% concentrate proportion (C_60_) in the ration (increasing from 35–60% during the first three weeks after parturition). Thus, four groups emerged: BCS_H_/C_60_ (n = 15), BCS_H_/C_35_ (n = 15), BCS_L_/C_60_ (n = 15), BCS_L_/C_35_ (n = 15). * Period (*p* < 0.001) for all variables, ^+^ Analyzed with first measured value from week 1 as covariate.

**Table 6:** Effect of body condition, C (Concentrate proportion in the diet) and period on change of back fat thickness and rib fat thickness (LSM) during period 1 (weeks 1–4 postpartum), period 2 (weeks 5–10 postpartum) and period 3 (weeks 11–17 postpartum) in the treatment groups. Negative values represent accretion of adipose tissue, positive values describe mobilization.

Item ^+^	Treatment ^§^				SEM ^#^	*p*-Value *					
	BCS_H_/C_60_ n = 15	BCS_H_/C_35_ n = 14	BCS_L_/C_60_ n = 15	BCS_L_/C_35_ n = 15		BCS	C	BCS × C	BCS × Period	C × Period	BCS × C × Period
Back fat thickness, cm/day											
Period 1	0.14	0.15	0.07	0.10	0.02	0.750	0.384	0.563	0.003	0.549	0.819
Period 2	−0.02	0.00	0.01	0.03	0.02						
Period 3	−0.01	−0.02	0.01	0.01	0.02						
Rib fat thickness, cm/day											
Period 1	0.04	0.07	0.09	0.09	0.03	0.136	0.567	0.881	0.903	0.809	0.827
Period 2	0.01	0.01	0.02	0.04	0.03						
Period 3	−0.05	−0.05	−0.02	−0.02	0.03						

^§^ Before calving cows were classified in high and low body condition score (BCS)-groups (BCS_H_, BCS_L_), after calving both groups were divided again, each into a group with 35% concentrate proportion (C_35_) and a group with 60% concentrate proportion (C_60_) in the ration (increasing from 35–60% during the first three weeks after parturition). Thus, four groups emerged: BCS_H_/C_60_ (n = 15), BCS_H_/C_35_ (n = 15), BCS_L_/C_60_ (n = 15), BCS_L_/C_35_ (n = 15). Values are presented as LSMeans, * Period (*p* < 0.001) for all variables, ^+^ Analyzed with first value measured before calving as covariate, ^#^ Pooled standard error of means.

## Appendix A

**Table A1:** Ultrasonic measuring points and its descriptions.

Measuring Points	Description
R12 ^+^	Subcutaneous fat over 12th rib
AW1b ^§^	Distance from skin to distal muscle margin above the peritoneum at the point of interception of a vertical line through the last lumbar vertebra and a horizontal line through the patella
AW3b ^§^	Distance from the skin to the distal muscle margin above the peritoneum at the center of the paralumbar fossa
AW3c ^§^	Thickness of the abdominal wall at the center of the paralumbar fossa
KD3b *	Distance from the skin to the peritoneum in the intertransverse space directly cranial to intertransverse space where the caudal pole of the kidney is visible
KD2c *	Distance from the skin to the distal kidney margin in the intertransverse space directly cranial to KD2c

^+^ R = rib, ^§^ AW = abdominal wall, * KD = kidney.

**Table A2:** Effects of body condition, concentrate proportion in the diet (C) and period on glucose and triglyceride concentrations in blood serum (LSM) during period 1 (weeks 1–4 postpartum), period 2 (weeks 5–10 postpartum) and period 3 (weeks 11–17 postpartum) in the treatment groups.

Item ^+^	Treatment ^§^				SEM ^#^	*p*-Value *					
	BCS_H_/C_60_ n = 15	BCS_H_/C_35_ n = 15	BCS_L_/C_60_ n = 15	BCS_L_/C_35_ n = 15		BCS	C	BCS × C	BCS × Period	C × Period	BCS × C × Period
Glucose, mmol/L											
Period 1	2.68	2.72	2.63	2.71	0.12	0.439	0.609	0.276	0.446	0.425	0.625
Period 2	3.06	2.99	2.85	2.88							
Period 3	3.23	2.88	3.04	3.07							
Triglycerides, mmol/L											
Period 1	0.13	0.13	0.12	0.13	0.01	0.066	0.044	0.149	0.034	0.151	0.077
Period 2	0.15	0.16	0.16	0.16							
Period 3	0.17	0.24	0.16	0.16							

^§^ Before calving cows were classified in high and low body condition score (BCS)-groups (BCS_H_, BCS_L_), after calving both groups were divided again, each into a group with 35% concentrate proportion (C_35_) and a group with 60% concentrate proportion (C_60_) in the ration (increasing from 35–60% during the first three weeks after parturition). Thus, four groups emerged: BCS_H_/C_60_ (n = 15), BCS_H_/C_35_ (n = 15), BCS_L_/C_60_ (n = 15), BCS_L_/C_35_ (n = 15). Values are presented as LSMeans, * Period (*p* < 0.001) for all variables, ^+^ Analyzed with first measured value before calving as covariate, ^#^ Pooled standard error of means.

**Table A3:** Effects of body condition, concentrate proportion in the diet (C) and period on efficiency parameters (LSM) during period 1 (weeks 1–4 postpartum), period 2 (weeks 5–10 postpartum) and period 3 (weeks 11–17 postpartum) in the treatment groups.

Item ^+^	Treatment ^§^				SEM ^#^
	BCS_H_/C_60_ n = 15	BCS_H_/C_35_ n = 15	BCS_L_/C_60_ n = 15	BCS_L_/C_35_ n = 15	
Feed efficiency ^†^, kg/kg					
Period 1	1.93	2.27	1.92	2.11	0.14
Period 2	1.58	1.84	1.54	1.81	0.14
Period 3	1.66	2.07	1.56	1.71	
Energy conversion efficiency ^#^, MJ/MJ NE_L_					
Period 1	0.88	1.04	0.89	0.97	0.03
Period 2	0.71	0.85	0.70	0.84	
Period 3	0.73	0.80	0.70	0.79	
Metabolic efficiency ^‡^, MJ NE_l_/kg body weight^0.75^					
Period 1	0.17 ^c^	0.00 ^c^	0.16 ^c^	0.07 ^c^	0.03
Period 2	0.38 ^b,AB^	0.20 ^b,CD^	0.43 ^b,A^	0.22 ^b,C^	
Period 3	0.35 ^a,A^	0.30 ^a,B^	0.42 ^a,A^	0.28 ^a,AB^	
Residual energy intake ^¶^, MJ NE_L_					
Period 1	27.9 ^a,A^	7.5 ^a,B^	21.7 ^a,A^	10.7 ^a,B^	3.8
Period 2	18.3 ^b,A^	−5.5 ^b,B^	17.1 ^b,A^	−3.3 ^b,B^	
Period 3	5.5 ^c,A^	−5.2 ^c,B^	6.7 ^c,A^	−6.5 ^c,B^	

^a,b^ Means with different superscripts differ within columns, ^A,B^ Means with different superscripts differ within row, ^§^ Before calving cows were classified in high and low body condition score (BCS)-groups (BCS_H_, BCS_L_), after calving both groups were divided again, each into a group with 35% concentrate proportion (C_35_) and a group with 60% concentrate proportion (C_60_) in the ration (increasing from 35–60% during the first three weeks after parturition). Thus, four groups emerged: BCS_H_/C_60_ (n = 15), BCS_H_/C_35_ (n = 15), BCS_L_/C_60_ (n = 15), BCS_L_/C_35_ (n = 15). Values are presented as LSMeans, ^+^ Analyzed with first measured value from week 1 as covariate, ^#^ Pooled standard error of means, ^†^ Feed efficiency (FE) = energy-corrected milk, kg/DMI, kg, ^#^ energy conversion efficiency (ECE) = energy excretion with milk, MJ/energy intake, MJ NE_L_, ^‡^ Metabolic efficiency (MEff) = (energy intake, MJ NE_L_ − energy in milk, MJ)/body weight^0.75^, kg, ^¶^ Residual energy intake (REI) = energy intake, MJ NE_L_ − expected energy intake, MJ NE_L_.

**Table A4:** *p*-values of effects of body condition, concentrate proportion in the diet (C), period and interaction between them on efficiency parameters.

Item ^+^	*p*-Value *					
	BCS	C	BCS × C	BCS × Period	C × Period	BCS × C × Period
Feed efficiency ^†^, kg/kg	0.240	0.009	0.509	0.491	0.997	0.718
Energy conversion efficiency ^#^, MJ/MJ NE_L_	0.291	<0.001	0.617	0.718	0.190	0.310
Metabolic efficiency ^‡^, MJ NE_l_/kg body weight^0.75^	0.182	<0.001	0.672	0.787	<0.001	0.002
Residual energy intake ^¶^, MJ NE_L_	0.920	<0.001	0.629	0.771	<0.001	0.04

Before calving cows were classified in high and low body condition score (BCS)-groups (BCS_H_, BCS_L_), after calving both groups were divided again, each into a group with 35% concentrate proportion (C_35_) and a group with 60% concentrate proportion (C_60_) in the ration (increasing from 35–60% during the first three weeks after parturition). Thus, four groups emerged: BCS_H_/C_60_ (n = 15), BCS_H_/C_35_ (n = 15), BCS_L_/C_60_ (n = 15), BCS_L_/C_35_ (n = 15), * Period (*p* < 0.001) for all variables, ^+^ Analyzed with first measured value from week 1 as covariate, ^†^ Feed efficiency (FE) = energy-corrected milk, kg/DMI, kg, ^#^ energy conversion efficiency (ECE) = energy excretion with milk, MJ/energy intake, MJ NE_L_, ^‡^ Metabolic efficiency (MEff) = (energy intake, MJ NE_L_ − energy in milk, MJ)/body weight^0.75^, kg, ^¶^ Residual energy intake (REI) = energy intake, MJ NE_L_ − expected energy intake, MJ NE_L_.
